# Supplementary material for: Novel Driver Strength Index highlights important cancer genes in TCGA PanCanAtlas patients
Source: PeerJ. 2022 Aug 11;10:e13860. doi: 10.7717/peerj.13860 (PMC9375969; doi:10.7717/peerj.13860)
Supplement: Supplemental Information 2 [file peerj-10-13860-s002.zip › COHORTS/genes plots/cohorts/2021_8_16_14_9_distribution_cohorts_PRAD.pdf]

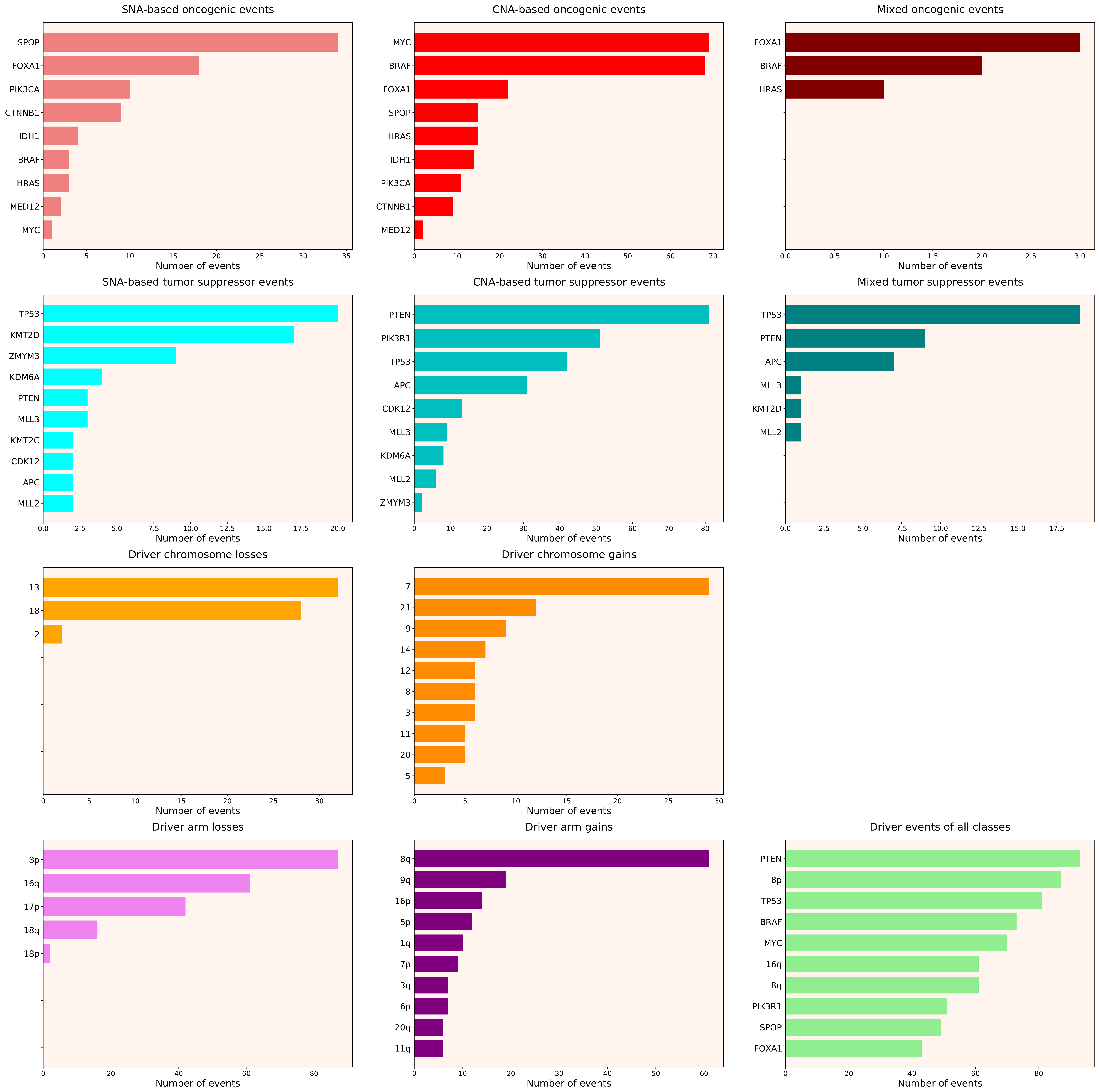
SNA-based oncogenic events
SPOP
FOXA1
PIK3CA
CTNNB1
IDH1
BRAF
HRAS
MED12
MYC
Number of events
CNA-based oncogenic events
MYC
BRAF
FOXA1
SPOP
HRAS
IDH1
PIK3CA
CTNNB1
MED12
Number of events
Mixed oncogenic events
FOXA1
BRAF
HRAS
Number of events
SNA-based tumor suppressor events
TP53
KMT2D
ZMYM3
KDM6A
PTEN
MLL3
KMT2C
CDK12
APC
MLL2
Number of events
CNA-based tumor suppressor events
PTEN
PIK3R1
TP53
APC
CDK12
MLL3
KDM6A
MLL2
ZMYM3
Number of events
Mixed tumor suppressor events
TP53
PTEN
APC
MLL3
KMT2D
MLL2
Number of events
Driver chromosome losses
13
18
2
Number of events
Driver chromosome gains
7
21
9
14
12
8
3
11
20
5
Number of events
Driver arm losses
8p
16q
17p
18q
18p
Number of events
Driver arm gains
8q
9q
16p
5p
1q
7p
3q
6p
20q
11q
Number of events
Driver events of all classes
PTEN
8p
TP53
BRAF
MYC
16q
8q
PIK3R1
SPOP
FOXA1
Number of events
